# Ethical Issues in Environmental Health Research Related to Public Health Emergencies: Reflections on the GuLF STUDY

**DOI:** 10.1289/ehp.1509889

**Published:** 2015-09-01

**Authors:** David B. Resnik, Aubrey K. Miller, Richard K. Kwok, Lawrence S. Engel, Dale P. Sandler

**Affiliations:** 1 Bioethics Program,; 2 Office of the Director, and; 3 Epidemiology Branch, National Institute of Environmental Health Sciences, National Institutes of Health, Research Triangle Park, North Carolina, USA; 4 Department of Epidemiology, Gillings School of Public Health, University of North Carolina at Chapel Hill, Chapel Hill, North Carolina, USA

## Abstract

Health research in the context of an environmental disaster with implications for public health raises challenging ethical issues. This article explores ethical issues that arose in the Gulf Long-term Follow-up Study (GuLF STUDY) and provides guidance for future research. Ethical issues encountered by GuLF STUDY investigators included *a*) minimizing risks and promoting benefits to participants, *b*) obtaining valid informed consent, *c*) providing financial compensation to participants, *d*) working with vulnerable participants, *e*) protecting participant confidentiality, *f*) addressing conflicts of interest, *g*) dealing with legal implications of research, and *h*) obtaining expeditious review from the institutional review board (IRB), community groups, and other committees. To ensure that ethical issues are handled properly, it is important for investigators to work closely with IRBs during the development and implementation of research and to consult with groups representing the community. Researchers should consider developing protocols, consent forms, survey instruments, and other documents prior to the advent of a public health emergency to allow for adequate and timely review by constituents. When an emergency arises, these materials can be quickly modified to take into account unique circumstances and implementation details.

## Introduction

Biomedical research can play a vital role in the response to public health emergencies such as natural disasters, disease epidemics, or terrorism (National Biodefense Science Board 2011; Ball 2013; Manuel 2001; Schwartz 2005). Based on lessons learned from the Gulf oil spill, Hurricane Sandy, and other similar disasters, Nicole Lurie (Assistant Secretary for Preparedness and Response), Francis S. Collins [Director of the National Institutes of Health (NIH)], and Thomas R. Frieden (Director of the Centers for Disease Control and Prevention) outlined the need for improved national capabilities to perform critical health research during disasters and other emergencies (Lurie et al. 2013) noting that research conducted during and after a public health emergency can provide critical knowledge that supports recovery efforts and improves capacity to deal with future emergencies. For example, research conducted in response to the Ebola outbreak in West Africa in 2014 has provided public health officials and the general public with additional information about virus transmission, mitigation of health risks, and appropriate measures to protect workers and prevent the spread of disease (IOM 2015b). In addition, the clinical trials conducted during the outbreak may lead to the development of treatments and vaccines (Cox et al. 2014). Observational and clinical research can help identify risk factors that impact the long-term health of people affected by public health emergencies and contribute to community and individual resiliency, susceptibility, or vulnerability.

Although timely research of populations affected by public health emergencies has been identified as a priority for disaster preparedness, response, and recovery, many challenges remain, including the need to address a number of important ethical issues prior to the onset of emergencies (IOM 2015a). In this article, we consider some of these ethical issues by drawing on insights gained from the Gulf Long-term Follow-up Study (GuLF STUDY; http://www.niehs.nih.gov/research/atniehs/labs/epi/studies/gulfstudy/).

## Background on the Gulf Oil Spill

On April 20, 2010, an explosion occurred aboard the *Deepwater Horizon* oil rig, which was located 49 miles off the Louisiana coast. Eleven people died in the explosion and in the subsequent sinking of the rig, which also damaged the oil well and led to the release of nearly 5 million barrels of oil into the Gulf of Mexico––making it the largest maritime spill in U.S. history (National Commission on the BP Deepwater Horizon Oil Spill and Offshore Drilling 2011). Additionally, about 1.84 million gallons of chemical dispersants were used to remediate the spill (Castranova 2011), which affected hundreds of miles of shoreline along the Gulf Coast of the United States between Florida and Louisiana. Cleanup workers used floating booms and skimmers to contain and collect the oil, sorbents to absorb it, and dispersants to break it up. Approximately 150,000 individuals from around the world participated in the cleanup effort, but most were from Louisiana, Mississippi, Alabama, and Florida. Local workers were mainly hired by contractors for British Petroluem (BP), which was the lessee for the *Deepwater Horizon* oil rig; others were sent by state or federal agencies. The well was capped on July 15, 2010, but cleanup work continued for many months (National Commission on the BP Deepwater Horizon Oil Spill and Offshore Drilling 2011).

## The GuLF STUDY

The White House and the Congress were eager to mount a quick response to the *Deepwater Horizon* oil spill, and they instructed federal agencies to act accordingly. Most of the response efforts involved the coordination and support of environmental cleanup and restoration activities, but some of the efforts included environmental health research. As part of the response by the NIH to the Gulf oil spill, intramural and extramural investigators from the National Institute of Environmental Health Sciences (NIEHS) and other NIH Institutes and Centers partnered with workers and numerous community groups in an array of research efforts to better understand the health impacts of the spill. The planning of the GuLF STUDY, which was the largest and earliest of these efforts, began in June 2010.

The primary objective of the GuLF STUDY is to investigate the potential short- and long-term health impacts associated with cleanup of the *Deepwater Horizon* oil spill. Secondary objectives include examining biomarkers such as genetic damage and alterations of gene expression in humans, which indicate the potential for adverse human health effects, and creating a resource for research on specific hypotheses or subgroups of interest within the study (Sandler et al. 2014). Cleanup workers, depending on their activities, may have been exposed to toxic chemicals found in crude oil and chemical dispersants, including volatile organic compounds, polycyclic aromatic hydrocarbons, hydrogen sulfide, 2-butoxyethanol, propylene glycol, and sulfonic acid salts. The physical and mental health of the workers may have also been affected by a variety of stressors related to the cleanup effort and economic disruption from the spill. Health outcomes of concern include changes in respiratory, cardiovascular, hematologic, and other physiologic functions; cancer; and mental health (Sandler et al. 2014).

The GuLF STUDY recruited approximately 33,000 adults who completed the safety training required to participate in the cleanup work. Most were hired to perform a variety of cleanup-related jobs. Those who were not hired—largely residents living in areas affected by the spill—represent an occupationally unexposed comparison population. In addition to an enrollment interview that was completed by all participants between March 2011 and March 2013, the study included home visits that generally occurred within 2 months after the enrollment interview. Approximately 11,200 participants residing in the Gulf states (i.e., Texas, Louisiana, Mississippi, Alabama, and Florida) completed the home visit. Blood, urine, hair, and toenail samples were collected and pulmonary function and blood pressure were measured during the home visits and at the health clinics. A comprehensive clinical examination is also being completed on as many as 4,000 participants who live within driving distance of health clinics in Mobile, Alabama and New Orleans, Louisiana.

The study participants will be followed for at least 10 years. Because most workers were not monitored for chemical exposures during cleanup operations, exposures are being reconstructed from available individual and environmental monitoring data, self-described characteristics of cleanup tasks, and work locations and times (Sandler et al. 2014).

Investigators and staff have made concerted efforts to reach out to the local communities concerning study design and implementation. Since September 2010, investigators have met with representatives from state and local health departments, advocacy and occupational groups representing workers involved in the cleanup, businesses, universities, and cultural and religious organizations. They have also formed a community advisory board with representatives from these different groups and have sought to employ individuals from affected communities to carry out the home visits and related study activities (e.g., making follow-up calls, handing out brochures, providing translation services) (Sandler et al. 2014).

Before considering the ethical issues related to the GuLF STUDY, it is important to be mindful of some important characteristics of the study population:

Many of the participants have suffered from psychological stress, depression, or trauma as a result of the oil spill’s impact on their well-being, community, local economy, and environment (Grattan et al. 2011).
Many of the participants are from low-socioeconomic positions that can adversely impact their health. For example, areas affected by the oil spill have some of the highest rates of poverty and unemployment and the lowest rates of access to health care in the United States (http://www.census.gov/hhes/www/cpstables/032014/pov/pov46_000.htm).
The region affected by the oil spill is culturally, ethnically, and linguistically diverse. Other than English, the languages spoken by the participants include Creole, Spanish, and Vietnamese (Sandler et al. 2014).
Many of the participants were transient—often living in group housing situations at the time of the study and relocating frequently—making it difficult for the study investigators to contact them for enrollment, for the baseline visit, or for follow-up (Sandler et al. 2014).
Many of the individuals in the coastal communities affected by the oil spill were also personally impacted by previous disasters such as Hurricanes Katrina and Rita (Kessler et al. 2008) and remain suspicious of governmental interventions.

## Discussion

*Risks and benefits.* Research regulations of the U.S. Department of Health and Human Services (DHHS) require that the risks to human subjects are minimized and reasonable in relation to the anticipated benefits to the participants and the knowledge to be gained (DHHS 2009). The observational GuLF STUDY involves no medical or environmental interventions. The main risks of the study include the potential for bruising or infection at the site where blood is drawn, some coughing or lightheadedness during pulmonary function testing, the risk of psychological stress from answering survey questions related to mental health or substance abuse, and the inadvertent loss of confidentiality. The NIEHS Institutional Review Board (IRB) determined that the GuLF STUDY presented no more than minimal risks to participants. The informed consent document describes the risks of the study for participants (https://gulfstudy.nih.gov/en/Consent_Form_Summary_Sheet_Clean_508%20Compliant.pdf).

Investigators were told by community representatives and local researchers that it would be important to provide tangible benefits to the participants to help ensure successful enrollment. Despite the implementation of the Affordable Care Act, a high percentage of study participants do not have private health insurance, nor are they covered by Medicaid or similar programs. Many of the participants were angry with BP and frustrated with what they perceived as a lack of government response to the oil spill. Some community activists urged researchers to provide health care rather than or in addition to health research. Although providing health care is outside the scope of NIH’s responsibility as a research organization, investigators responded to these concerns by developing, in collaboration with the Health Resources Services Administration (HRSA), the Substance Abuse and Mental Health Services Administration (SAMHSA), and local health departments, detailed information on health care providers in the region and on low- or no-cost health care options that they shared with participants. The study investigators also developed area-specific listings of health and mental health care providers for referral of participants in need of health services. Study staff made direct health care referrals by providing information on federally qualified health centers in the area, including arranging for free care in exceptional situations.

The study also has provided participants with potentially useful health information, including the results of clinical tests and medical examinations such as blood pressure, pulmonary function, body mass index, and urine glucose levels when available. Participants with abnormal findings were advised to consult a health care provider and were given referrals if needed. They received individual and summary results. The individual-level results (e.g., clinical blood chemistries or current chemical levels in a subset) provided to participants included information to help them understand their test results. For example, individual-level results explained the normal ranges for the GuLF STUDY population and, when pertinent, compared the findings with a nationwide sample such as the National Health and Nutrition Examination Survey. The reports also indicate when clinical implications are unclear or unknown. Participants who were selected for the baseline analysis of the clinical chemistries or current chemical exposures received reports of test results performed by certified laboratories with explanations of their results. All participants also received summaries of study findings via the study website and through newsletters or mailings (http://www.niehs.nih.gov/research/atniehs/labs/epi/studies/gulfstudy/publications/index.cfm/).

*Informed consent.* Federal research regulations require that investigators obtain informed consent from participants or their legal representatives (DHHS 2009). The informed consent process presented challenges to the study staff because some participants were suffering from stress, depression, trauma, or other mental health conditions that could compromise their ability to make informed decisions. Many participants are from low-socioeconomic positions, and some have limited English-language skills (Lange et al. 2013). Many home visits were conducted in group housing and other challenging settings. To address these issues, research staff members were trained on how to conduct consent discussions (Sandler et al. 2014) in circumstances that present difficulties for participants. Potentially eligible participants were first contacted by mail and given at least 2 weeks to opt out of the study. The mailing contained a study brochure (http://www.niehs.nih.gov/research/atniehs/labs/epi/studies/gulfstudy/publications/gulf_study_brochure.pdf), including a description of the research and text highlighting key consent elements, as well as information on how to contact study staff to answer questions. Initial enrollment interviews were conducted by telephone. Since the completion of a phone interview was taken to imply consent, and to meet requirements of the U.S. DHHS Certificate of Confidentiality (discussed in the “Confidentiality” section of this article), the interviewers began with a lengthy explanation of the potential risks and benefits of the study along with other information needed to obtain informed consent. Because most of the participants used cell phones, some of which had limited minutes, this lengthy script (approximately five minutes in a 30–60 minute interview) proved to be a barrier to participation—with many break-offs during this stage of the study. Of the 58,923 potential participants reached by phone, 22,572 (38.3%) either contacted the study center to opt out of the study or hung up on the interviewer before the consent scripts could be administered. A total of 36,351 individuals listened to the consent script; of these, 2,395 (6.6%) refused to enroll in the study. Home visits included written informed consent using a document that often had to be read to participants. Along with a copy of the consent document, participants received a guide that highlighted the consent form and a frequently asked questions document (https://gulfstudy.nih.gov/en/faqs.html) to provide them with clear and concise information about the study. Consent documents and other study materials were translated into Spanish and Vietnamese, and individuals who were fluent in these languages were included in the home visits. Community groups were also consulted on how best to approach consent and recruitment. These groups provided valuable feedback on recruitment methods, materials and language used, and suggested revisions as appropriate; they also hosted community forums where potential participants learned more about the study.

*Participant financial compensation.* One recurring theme from community meetings and reviews by expert panels during the study design phase was the need for financial remuneration for the participants. Many experienced investigators from the region indicated that individuals would not participate without compensation. Furthermore, some believed it was unethical to ask vulnerable injured parties, such as those impacted by the spill, to participate with no tangible benefit. However, excessive financial compensation may constitute an undue inducement to participate in research, especially for participants who are from low-socioeconomic positions (Grady 2005). Given the size of the study cohort, remuneration was initially considered to be cost prohibitive, at least for the larger cohort participating in the telephone interviews. Nonetheless, as challenges to locating and recruiting participants became clear, the investigators worked with the IRB to devise an incentive plan that included drawings for a $500.00 gift card for every 500 participants who enrolled in the study, as well as drawings among early responders (e.g., the first 500 who called in to complete their interview and/or schedule their home examination). For those participating in the home visits, each person received a $50.00 gift card as remuneration for time spent completing the examination and providing samples regardless of whether they completed all aspects of the exam or not.

*Vulnerable participants.* Federal research regulations require that studies include safeguards to protect subjects who may be vulnerable to coercion or undue influence. The regulations also include special protections for children, prisoners, pregnant women, fetuses, and neonates (DHHS 2009). Many of the participants in the study could be considered vulnerable because of mental health issues, socioeconomic deprivation, minority status, language barriers, or previous experiences with other disasters in their community (Kessler et al. 2008). As mentioned previously, the study includes procedures to ensure that participants from vulnerable groups can provide valid informed consent. Although the study does not pose any significant risks to the fetus, pregnant women were excluded from pulmonary function testing until three months postpartum to minimize risks. The study was not designed to enroll prisoners, children, or neonates.

*Confidentiality.* Federal regulations require that research involving human subjects include appropriate measures to protect privacy and confidentiality (DHHS 2009). The GuLF STUDY includes a variety of measures to protect the confidentiality of participants, including a Certificate of Confidentiality (http://www.niehs.nih.gov/research/atniehs/labs/epi/studies/gulfstudy/publications/gulf_study_certificate_of_confidentiality.pdf) issued by the U.S. DHHS that investigators can use to resist requests for access to data. Biological samples (e.g., blood and hair) and data will be shared with other researchers only with IRB approval. To obtain samples or data, other researchers must agree to maintain confidentiality, use the samples or data only for approved research purposes, and not attempt to identify individuals (Sandler et al. 2012).

The most significant confidentiality issues involved the reporting of suspected child, elder, or spousal abuse or threats of harm to self or others that were discovered during telephone calls or home visits. The need to have appropriate procedures and training in place was anticipated due to information obtained from early meetings with community and local health agencies and from research on prior disasters where mental health issues were paramount (Child Welfare Information Gateway 2014). Study staff members were trained on how to handle situations that might require reporting to social services, the police, or other authorities. Participants were told during the informed consent process that study staff might make these reports. Interviewers and home examiners have, in fact, encountered situations where participants appeared to be in danger from violent housemates and instances in which participants or others threatened to harm other people or themselves. These incidents were handled by notifying the appropriate local authorities when necessary, connecting participants directly with helplines or mental health services, and reporting them to the IRB.

*Conflict of interest.* Since real or apparent financial conflicts of interest can undermine the integrity and trustworthiness of scientific research (IOM 2009), it was important for the GuLF STUDY to address such issues. Funding for the GuLF STUDY is largely provided by the NIH Office of the Director via the NIH Common Fund and by the NIEHS. Early on, BP provided a $10 million gift to the NIH for health research conducted in the states impacted by the *Deepwater Horizon* oil spill (NIEHS 2010). The NIH leadership allocated a portion of these funds to the GuLF STUDY. At least $6 million out of the $40 million spent so far on the study has come from BP. To avoid any real or apparent financial conflict of interest, steps have been taken to ensure that BP has no involvement in designing or implementing the study, analyzing the data, or interpreting and disseminating the results. BP’s only involvement, disclosed to participants during the consent process (http://www.niehs.nih.gov/research/atniehs/labs/epi/studies/gulfstudy/publications/gulf_study_informed_consent_form.pdf), was its early gift to the NIH and the provision of access to needed exposure monitoring and workforce information.

*Legal climate.* Many people living in the area affected by the oil spill are pursuing, or are considering pursuing, litigation against BP. The prospect of lawsuits against BP has had a potential impact on recruitment and disclosure of information in the GuLF Study. Lawyers, for example, may tell clients not to participate in research lest they create a record that can be used to challenge any possible health claims. If widespread, such advice can undermine participation and threaten study validity through low-response rates. Alternatively, individuals who are experiencing health symptoms may have an incentive to participate because they want to create a record of harm for use in litigation. When questions arose, study staff attempted to explain to the participants the limited value of study data for lawsuits as well as steps taken to prevent unauthorized release of data. These explanations occurred informally on an ad hoc basis. Nevertheless, procedures were established with the NIH Freedom of Information Act (FOIA) Office to handle requests for information from participants or their legal representatives and from BP. Efforts also are underway to work with the legal community (e.g., personal injury attorneys) to clarify what information the GuLF STUDY does and does not collect.

*IRB oversight.* GuLF STUDY investigators realized from the outset that it was important for research activities to commence as soon as possible to capture relevant biomarkers, avoid degradation of environmental samples, and minimize loss to follow-up of the workers and loss of recall of relevant information on cleanup activities. As noted earlier, there was also considerable political and public pressure to mount a rapid response to the spill. The NIEHS IRB was consulted early in the design process and took steps to help the investigators obtain approval in a timely fashion while ensuring that adequate protections were in place for human participants. GuLF STUDY investigators communicated closely with IRB staff to discuss proposed approaches and to share drafts of the protocol, consent form, and other documents with the IRB prior to submission; they also obtained helpful feedback from the IRB chair, vice-chair, and staff. Because of the unique challenges related to the legal climate surrounding such a large-scale environmental disaster, the study team also involved NIH legal counsel in the review of proposed consent language and recruitment materials. To help facilitate a timely study start, the IRB scheduled a special session to review the GuLF STUDY. A full packet (e.g., the study protocol, consent form, questionnaires, and data collection forms) was submitted to the IRB in October after the study proposal was reviewed in September by a panel convened by the Institute of Medicine at the request of Francis S. Collins, Director, NIH. The IRB made a number of stipulations concerning the proposed study at its November 2010 meeting. The investigators responded to these requirements, and the IRB gave its final approval in late December 2010. Recruitment began shortly thereafter and the first participants were enrolled in February 2011. This time frame was considerably shorter than that for other studies of this magnitude and complexity and required IRB members to set aside other work to review materials as they became available and for investigators to work evenings and weekends to meet deadlines. The investigators have continued to submit amendments to the IRB as they have refined study documents and procedures and added substudies (Sandler et al. 2014).

Despite efforts to accelerate the review process, data collection did not begin until eight months after the spill occurred. Although scientific, ethical, and administrative reviews contributed to this delay, most of the delays were due to the time required for project development, including

Gathering information needed to design a scientifically valid study.
Drafting the protocol, consent forms, and questionnaires.
Determining mechanisms for identifying those who were engaged in the cleanup effort.
Securing access to records.
Establishing partnerships with relevant community and governmental groups.

Conducting some of these activities prior to a public health emergency may help to reduce delays in future studies.

## Conclusions

Environmental health research related to disasters and other public health emergencies raises challenging ethical issues that need to be addressed beforehand, including

Minimizing risks and promoting benefits to participants.
Obtaining valid informed consent.
Providing financial compensation to participants.
Working with vulnerable participants.
Protecting participant confidentiality.
Addressing conflicts of interest.
Dealing with legal implications of research.
Obtaining review from the IRB, community groups, and other committees (e.g., scientific review committees).

To ensure that these issues are handled properly, it is important for investigators to work closely with the IRB during the development and implementation of research and to consult with groups representing the community and government agencies involved in emergency response. To promote timely IRB review, researchers may want to work with their IRBs prior to the onset of public health emergencies to develop standardized modular protocols, consent forms, surveys, and related documents (e.g., instruction booklets and brochures for participants, training materials for research teams). When an emergency arises, these materials can be modified quickly to take into account any relevant or unique circumstances, including the population to be studied and the specific exposures and expected health consequences. Such an approach would ensure adequate review by IRBs and other groups of complex ethical issues without jeopardizing rapid response to an emerging public health disaster. In response to these challenges the NIEHS has spearheaded the development of a new Disaster Research Response Project to facilitate the incorporation of “disaster science” into national response and recovery efforts. The following key components of this project include:

Improving accessibility to health data collection tools and research protocols that can be quickly modified and implemented when an emergency occurs.
Engaging diverse public and private stakeholders.
Fostering the development of a trained cadre of academic researchers who can collect critical information in the immediate postdisaster environment without interfering with the emergency response (NIH 2015).
