# Erratum: “Ethical Issues in Environmental Health Research Related to Public Health Emergencies: Reflections on the GuLF STUDY”

**DOI:** 10.1289/ehp.124-A29

**Published:** 2016-02-01

**Authors:** David B. Resnik, Aubrey K. Miller, Richard K. Kwok, Lawrence S. Engel, Dale P. Sandler

Environ Health Perspect 123(9):A227–A231 (2015), http://dx.doi.org/10.1289/ehp.1509889

In the acknowledgments of this article, the authors neglected to recognize the contributions of Joan P. Packenham, Director of the National Institute of Environmental Health Sciences (NIEHS) Office of Human Research Compliance, Clinical Research Program (CRP) and Vice-Chair of the NIEHS Institutional Review Board (IRB); Jane M. Lambert, NIEHS CRP IRB Administrator; and Craig Wladyka, NIEHS, CRP, IRB Protocol Coordinator, for their roles in the review and oversight of the GuLF STUDY. The comments they made during the review process were incorporated into the study protocol and consent documents and played an important role in ensuring ethical and regulatory oversight of the study.

The authors regret this omission.

